# Asparaginase activity monitoring in pediatric acute lymphoblastic leukemia: A cross‐sectional nationwide study in Spain

**DOI:** 10.1002/cnr2.1729

**Published:** 2022-10-28

**Authors:** Álvaro Lassaletta, Fernando Gutiérrez, the PEGASO study investigators

**Affiliations:** ^1^ Pediatric Hematology‐Oncology Department Hospital Infantil Universitario Niño Jesús Madrid Spain; ^2^ Research Department, Pharmacy Department Complejo Hospitalario Universitario de Canarias Santa Cruz de Tenerife Spain

**Keywords:** acute lymphoblastic leukemia, asparaginase, asparagine, cross‐ sectional study, survey

## Abstract

**Background:**

A cross‐sectional nationwide study was designed to assess national compliance with international consensus/guidelines of monitoring asparaginase levels in children with acute lymphoblastic leukemia (ALL) treated with asparaginase in routine clinical practice.

**Methods:**

An ad hoc questionnaire was designed and completed by staff physicians from Hemato‐Oncology Units throughout Spain.

**Results:**

A total of 39 physicians (64% pediatricians) with a mean (SD) age 43.5 (7.9) years and 15.3 (17.6) years of professional activity participated in the study. They accounted for 90% of hospitals in which children with ALL are treated in Spain. A total of 19 participants (48.7%) reported that asparaginase levels were routinely monitored (own center in 2 cases [10.5%], another hospital in 17 cases [89.5%]). Asparaginase was not monitored in 51.3% of the cases, mostly (80%) because unavailability of testing. When asparaginase was monitored, 68% of participants reported that this was done in all asparaginase‐treated patients and 84% in all phases of the disease (induction, consolidation, re‐induction, maintenance) with a time interval of 7 days for the pegylated form, 48 h for *Erwinia* asparaginase and 14 days for maintenance with the pegylated form. All participants reported that they modified treatment according to results of testing, with a limit of total depletion of ≥100 IU/L. Levels <100 or 20 IU/L were considered indicative of hypersensitivity by 46% of physicians.

**Conclusion:**

There is still a gap between what is recommended and what is done in clinical practice, with more than 50% of centers not monitoring the level of asparaginase activity in pediatric ALL. Protocols for asparaginase testing in daily practice should be implemented.

## 1 INTRODUCTION

Acute lymphoblastic leukemia (ALL) is the most common childhood cancer, accounting for approximately of 25% of all cancer diagnoses among children younger than 15 years of age.^1^ The risk is highest in children under 5 years of age and then, declines slowly until the mid‐20s.^2^ Although ALL has one of the highest cure rates of all childhood cancers (overall survival of more than 80%), between 15% and 20% of patients who had achieved complete remission following initial multi‐agent chemotherapy will have a relapse.^3^ Targeting tumor cell metabolism has become an attractive form of therapy. Tumor cells often rely on an exogenous supply of amino acids and limiting the availability of this increased demand can be selectively lethal for the inability of cancer cells to sustain growth in the absence of a particular nutrient (auxotrophy).^4^

Asparagine synthetase catalyzes the synthesis of the non‐essential amino acid asparagine from aspartate and glutamine. Lack of asparagine synthetase protein expression is a hallmark of ALL blasts, which, therefore, are auxotrophic for asparagine.^5^ This peculiarity is the rationale for the use of bacterial L‐asparaginase for ALL therapy. Native L‐asparaginase derived from *Escherichia coli*, an enzyme isolated from *Erwinia chrysanthemi* and a pegylated form of the native *E. coli* are the three main types of L‐asparaginases. When properly used, there are no survival differences between the three types of asparaginases in ALL. Asparaginase blood levels are measured as a surrogate for asparagine depletion and resultant leukemic cell death.^6^ Full asparagine depletion and high asparaginase activity are both associated with improved outcomes in children and adult ALL populations.^7^, ^8^

Therapeutic drug monitoring is critical to detect silent inactivation of the drug due to anti‐asparaginase neutralizing antibodies.^9^, ^10^, ^11^ Although there is no universally accepted dose or treatment schedule for all patients, it has been generally recommended that ≥100 IU/L is an appropriate target trough level of asparaginase activity.^12^, ^13^, ^14^ Interpatient variability, pharmacokinetic differences in asparaginase formulations and host‐related factors can affect the enzymatic activity, so that to ensure optimal treatment and reduce toxicity, physicians should be aware of these sources of variability and be ready to make any necessary treatment adjustments.^15^, ^16^, ^17^

A failure to implement asparaginase drug monitoring may fail to recognize patients who warrant a switch to *Erwinia*. Therefore, therapeutic drug monitoring is particularly useful for optimizing asparaginase treatment, and when a tight pharmacological monitoring program is established, premedication could be implemented more broadly to minimize the risk of adverse reactions.^18^ However, data on the implementation of routine measurement of asparaginase levels in daily practice are scarce. In a survey study across every province in Canada, only 39.2% of pediatric oncologists routinely monitored asparaginase levels.^19^ The findings of a study in Brazil presented a worrisome scenario in which, despite provision of L‐asparaginase for all oncological centers in the country by the public health system, the use of laboratory tests to measure asparaginase activity was very limited.^20^ The present nationwide study was designed to assess monitoring of asparaginase levels in children with ALL treated with asparaginase in routine daily practice in Spain.

## 2 METHODS

### 2.1 Study design

A cross‐sectional, multicenter, non‐randomized, survey‐based nationwide study (PEGASO study) was designed in the setting of pediatric hemato‐oncology units throughout Spain. PEGASO is the acronym of Pegasparaginase Activity: Pursuit Solutions. The primary objective was to assess the use of therapeutic drug monitoring in children and adolescents with ALL treated with asparaginase in routine clinical practice. This information could be useful to identify gaps in the management of childhood ALL and to develop recommendations to ensure that therapeutic levels of asparaginases are consistently maintained for improving outcome. The study was based on a survey using an ad hoc questionnaire and was conducted over 2 months (May–June 2021). Since no patient data were collected, the study did not need to be submitted to a clinical research ethics committee and was exempt from the requirement for informed consent.

### 2.2 Participants and procedures

To carry out the study, a scientific committee was established with two specialists, one in pediatric hemato‐oncology, and the other in clinical research. The members of the scientific committee (Álvaro Lassaletta, Fernando Gutiérrez) were responsible for the development of the study protocol and the creation of the questionnaire. They also coordinated and supervised the conduct and progress of the study, including the review of the results and data analysis. The final questionnaire included four sections with data related to the participants (6 items), their working centers (2 items), clinical consultations (2 items) and monitorization of asparaginase activity levels (9 items). The questionnaire is described in the supplementary material.

Candidates for participation in the study were specialists in hematology and pediatrics who routinely treated children with ALL and provided care in public and private hospitals located nationwide, representing almost all centers available in Spain for the care of childhood ALL. They were recruited by means of an invitation leaflet, which detailed the characteristics of the study and provided them with the URL of the platform that included the study questionnaire and the user password. Consent to participate in the study was requested when accessing the questionnaire. Participation was anonymous and voluntary.

### 2.3 Statistical analysis

Recruitment of participants was non‐competitive and with a sample size of 50 investigators (centers), a 15% error margin for a confidence interval of 95% and heterogeneity of 50% was obtained. The descriptive analysis of data included frequencies and percentages for categorical variables, and mean and standard deviation (DE) for continuous variables. The data were analyzed with the SAS statistical program (Statistical Analysis Systems, SAS Institute, Cary, NC, USA) version 9.4 for Windows.

## 3 RESULTS

### 3.1 Characteristics of participants

All 39 specialists invited to participate in the study agreed and completed the survey. There were 10 men and 29 women, with a mean (SD) age of 43.5 (7.9) years and 15.3 (17.6) years of professional activity. A total of 25 (64.1%) participants were specialists in pediatrics, 9 in hematology, and the remaining 5 in hematology and pediatrics. The hospitals in which these specialists were working accounted for 90% of all centers in which children with ALL are treated in Spain. Participation in a training program and in a research program on ALL in the past 12 months was reported by 87.2% and 69.2% of participants, respectively. Also, 48.7% reported that they had been attended less than 25 pediatric patients with newly diagnosed cancer in the last year and 41% between 25 and 50. On the other hand, 23 participants (59%) visited between 5 and 15 patients with a new diagnosis of ALL in the last year.

### 3.2 Asparaginase activity levels monitoring

Data related to asparaginase levels monitoring are shown in Table 1. A total of 19 (48.7%) participants reported that asparaginase levels were routinely monitored, in their own center in 2 and in another center in 17. Asparaginase levels were not monitored in 51.3% of the institutions, mostly because testing was not available in the hospital (80%), although 95% would like access to asparaginase levels in their centers, with testing performed in the pharmacy or clinical laboratory services according to the opinion of 68.4% of participants.

**TABLE 1:** Results regarding asparaginase activity levels monitoring

Items of the questionnaire	Number (%)
Are asparaginase levels monitored in your center? (*n* = 39)	
Yes	19 (48,7)
In your own center	2 (10.5)
In another center	17 (89.5)
No	20 (51.3)
Reasons for not monitoring asparaginase levels (*n* = 20)	
Lack of access to the test in the hospital	16 (80.0)
I would like to send to another center, but I have no funding	1 (5.0)
Other^a^	2 (10.0)
Do not know/no answer	1 (5.0)
Would you like that asparaginase levels monitoring could be determined in your center? (*n* = 20)	
Yes	19 (95.0)
Do not know/no answer	1 (5.0)
In which service do you think that testing should be performed? (*n* = 19)	
Hematology	1 (5.3)
Pharmacy	7 (36.8)
Laboratory	6 (31.6)
Biochemistry	3 (15.8)
Do not know/no answer	2 (10.5)
Which patients are monitored for asparaginase levels? (*n* = 19)	
Patients with ALL	4 (21.1)
Patients with lymphoma	0
All patients treated with asparaginase	13 (68.4)
Other^b^	2 (10.5)
How long do you allow after the administration of asparaginase before testing activity levels? (*n* = 19)	
Pegylated asparaginase at 7 days and *Erwinia* asparaginase at 48 h, in case of maintenance with pegylated asparaginase at 14 days	16 (84.2)
At 14 days	1 (5.3)
At 7 days	2 (10.5)
At 48 h	0
How do you use asparaginase activity levels in decision making (*n* = 19)	
We do not modify treatment based on the result	0
We modify treatment according to the levels (switch to *Erwinia* asparaginase in case of allergy) or silent inactivation confirmed by activity levels)	19 (100.0)
Other^c^	0

^a^ Already approved and in process (*n* = 1), no support for the management of samples and no funding (*n* = 1).

^b^ Selected patients all of them with ALL (*n* = 1), patients with adverse reactions considering dose modification and patients with doubtful hypersensitivity reactions (*n* = 1).

^c^ Doses are not modified except in case of activity <100 UI/L in two assays (*n* = 1), switch to *Erwinia* asparaginase in case of allergy or silent inactivation, and in a timely manner, modifying doses in the presence of adverse events (*n* = 1).

**FIGURE 1:**
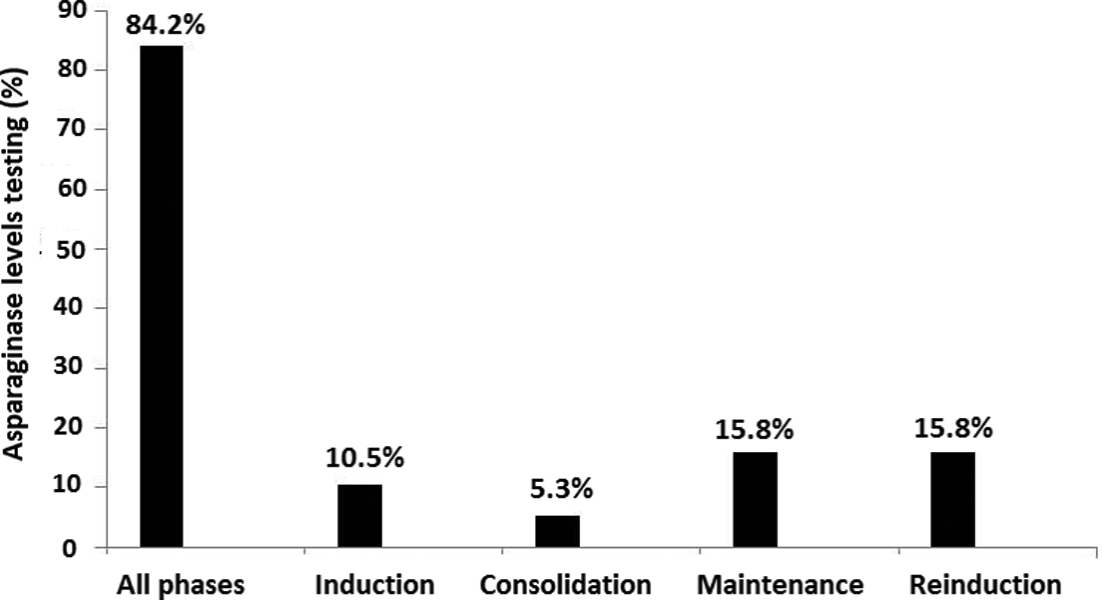
Measurement of asparaginase activity levels and treatment phases of ALL (multiple response).

**FIGURE 2:**
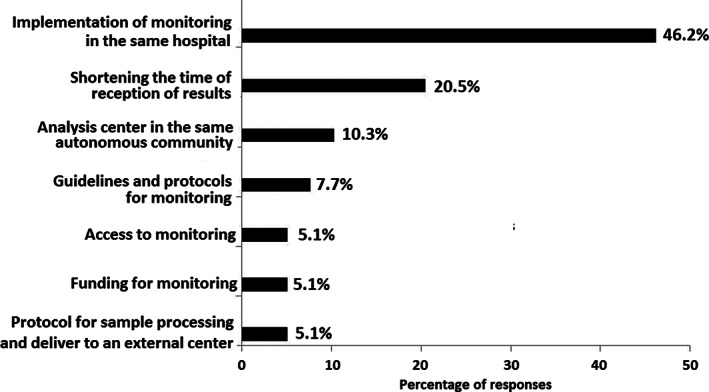
Main areas of improvement in asparaginase activity levels monitoring in the participant's center (multiple response).

In the group of 19 participants who reported that asparaginase levels were monitored, 68.4% reported that testing was performed in all asparaginase‐treated patients, 21.1% only in patients with ALL, and 10.5% in lymphoma patients or in other situations. As shown in Figure 1, asparaginase levels were monitored in all phases of the disease in 84.2% of cases and in the induction, maintenance, reinduction, and consolidation phases in 10.5%, 15.8%, 15.8% and 5.3% of cases, respectively. Also, 84.2% of participants reported that the length of time between starting the administration of asparaginase and activity levels monitoring was 7 days for pegylated asparaginase, 48 h for *Erwinia* asparaginase, and 14 days in patients on maintenance with pegylated asparaginase (Table 1). Moreover, 100% of participants modified treatment according to results of testing, switching to *Erwinia* asparaginase in case of allergy. None of the participants used premedication, and in case of a holiday, treatment was delayed only in case of using pegylated asparaginase (82.1%) (Table 2). A limit of total depletion of ≥100 IU/L was defined by 94.9% of participants, and levels of <100 or 20 IU/L were considered indicative of hypersensitivity by 46.2% of participants. After the appearance of symptoms of hypersensitivity, if asparaginase activity monitoring is available and activity has not been lost, 69.2% of participants considered that medication could not be changed (Table 2). Finally, as shown in Figure 2, main areas of improvement included implementation of asparaginase monitoring in the hospital, to reduce the length of time for the reception of results, and to develop guidelines/protocols for asparaginase activity levels monitoring.

**TABLE 2:** Results regarding the use asparaginase activity levels monitoring

Items of the questionnaire	Number (%)
In case of a holiday, do you delay the administration of asparaginase? (*n* = 39)	
Yes independently of which we were using	4 (10.3)
In case of pegylated asparaginase only	3 (7.7)
No	32 (82.1)
Do you use premedication in the administration of asparaginase (*n* = 39)	
Yes	0
No	39 (100.0)
What is the limit that you consider that is the target of total asparaginase depletion? (*n* = 39)	
≥50 IU/L	0
≥100 IU/L	37 (94.9)
≥200 IU/L	0
≥500 IU/L	1 (2.6)
Do not know/no answer	1 (2.6)
When do you consider that a patient presents an hypersensitivity reaction to pegylated asparaginase (headache, flushing, encephalopathic symptoms…) (*n* = 39)	
After confirmation asparaginase levels <100 IU/L or 20 IU/L (according to the time elapsed from treatment)	18 (46.2)
If symptoms reappeared in the next administration	3 (7.7)
In the presence of symptoms, the patient has an allergic reaction	10 (25.6)
Other^a^	7 (17.9)
Do not know/no answer	1 (2.6)
After the appearance of symptoms compatible with hypersensitivity … (*n* = 39)	
… a change of asparaginase should be always made	6 (15.4)
… I could not change it, if I have the asparaginase determination available, and I confirm that activity has not been lost	27 (69.2)
… try to administer asparaginase again with premedication	2 (5.1)
Other^b^	4 (10.3)

^a^ Suggestive symptoms of hypersensitivity; if symptoms have an immediate cause‐effect; after a suspicion of an allergic reaction we switch to *Erwinia*; only in case of mild unclear symptoms of hypersensitivity, we repeat the dose under hospital surveillance; the presence of symptoms means that the patient has an allergic reaction.

^b^ Depends on both type of reaction and severity; if type and severity of symptoms are quite evident and there is a clear cause‐effect relationship, I would switch to another asparaginase; in the presence of a minimum suspicion of hypersensitivity, we switch to *Erwinia* because asparaginase activity is not monitored.

## 4 DISCUSSION

The development of effective treatments and well‐designed clinical protocols have resulted in a dramatic increase of the long‐term outcome of ALL over the last few decades, with overall survival rates exceeding 90%, and without compromising the quality of life of survivors.^21^, ^22^ The bacterial‐derived enzymes, asparaginases, are among the drugs used in the treatment of ALL, with *E. coli* asparaginase, a pegylated form of the native *E. coli* asparaginase, and an enzyme isolated from *Erwinia* as the three main types. Other novel forms of recombinant *E. coli* asparaginase^23^ and a pegylated recombinant formulation of *Erwinia* asparaginase^24^ have been recently developed and are currently under investigation. A recombinant form of native *Erwinia* has now been FDA approved for use in the US (asparaginase erwinia chrysanthemi [recombinant]‐rywn, tradename Rylaze).^25^ However, although extensive clinical evidence supports the administration of asparaginase therapy in pediatric ALL and the benefit of intensive asparaginase regimens,^26^, ^27^ debate continues regarding the optimal dosing and schedule, duration of treatment, indication of front‐line or second‐line asparaginase therapy, or management of toxicities.^28^, ^29^

Although L‐asparaginase preparations are included in the pharmaceutical portfolio of public healthcare systems of many countries, there is little information on assessment of asparagine depletion in routine daily practice. The aim of this study was to collect data on the use of asparaginase activity monitoring in children with ALL treated with asparaginase preparations in the hemato‐oncology units of our country. Interestingly, the study collected real‐world data since the 39 specialists who participated in the study and completed the questionnaire were representative of 90% of hospitals providing care to children with ALL in Spain.

Different findings should be emphasized. One of the most important is the gap between what is recommended and what is done in clinical practice, with 51.3% of centers in which the level of asparaginase activity was not monitored in childhood ALL. On the other hand, among the specialists (48.7%) who reported the use of asparaginase activity monitoring, testing was available at their own institutions in only 10.5% of cases, having to rely on testing performed at external centers in the remaining 89.5% of cases. In fact, one of the areas of improvement was to implement asparaginase activity monitoring in their own centers reported by 46.2% of participants and to have available a laboratory analysis at the same geographical area reported by 10.3%.

The present results showing clear shortcomings in the routine assessment of asparaginase levels in the care of childhood ALL are consistent with results of a Canadian survey based on data from 51 pediatric hematology‐oncology specialists, in which only 39.2% of respondents reported routinely measuring asparaginase activity.^19^ In this survey, the most common reason for not measuring asparaginase levels was not knowing how to use levels clinically (25.5%), whereas in our study, lack of access to the test in the hospital was the main reason reported by 80% of participants. However, it is important to mention that laboratory methods are generally cheap and simple.

It has been established that a serum level of asparaginase of >100 IU/L corresponds to depletion of asparagine (i.e., below the limit of quantification), and in this respect, 95% of participants in our study correctly considered this limit as the target of total depletion. Also, about 90% of participants used asparaginase activity levels in decision‐making, switching from pegylated asparaginase to *Erwinia* asparaginase in case of allergy or silent inactivation confirmed by assessment of the therapeutic asparaginase level. Patients exhibiting clinical allergy symptoms to one formulation of asparaginase are typically switched to another product to ensure they receive the most efficacious treatment regimen as possible. However, silent inactivation lack of clinical symptoms and suboptimal depletion may result if asparaginase activity levels are not monitored. Although rare, a 7% silent inactivation was observed in UKALL 2003, with very similar dosing of pegylated asparaginase as in the Spanish ALL protocol.^1^, ^30^ The importance of therapeutic drug monitoring has been highlighted in different studies,^31^, ^32^ showing that asparaginase preparations are not readily interchangeable and identical doses may be associated with different enzyme activities.^33^ It has been suggested that a multifaceted approach based on creation of a multidisciplinary asparaginase allergy committee to review all hypersensitivity reactions, staff education on the management of asparaginase therapy, and implementation‐standardized guidelines for therapeutic drug monitoring can safely maintained patients on pegylated asparaginase and conserve switching to *Erwinia* for patients who need it.^34^ On the other hand, monitorization of asparaginase activity allows the use of premedication and, therefore, to reduce the probabilities of silent inactivation.

Within Spanish hospitals that do routinely measure asparaginase levels, there was consistency regarding the timing of levels, with 84.2% of participants reporting day 7 for pegylated asparaginase, 48 h for *Erwinia*, and 14 days in case of maintenance with pegylated asparaginase (in the intermediate risk of the National ALL protocol, there is a pegylated asparaginase intensification every 2 weeks). Also, 100% of participants indicated that they did not administer premedication before asparaginase therapy. However, premedication use and asparaginase level monitoring may contribute to prevent adverse events, drug substitutions, and may be cost‐effective in pediatric ALL.^35^, ^36^

Results of the present study should be interpreted taking into account the observational characteristics of the survey based on self‐reporting. However, the number of participants with solid experience in the management of pediatric cancer and accounting for 90% of hospitals in which children with ALL are treated in Spain, ensures that the survey was able to capture real‐world data of asparaginase activity monitoring in clinical practice.

In conclusion, this study shows that efforts on different perspectives including widespread implementation of specific protocols of therapeutic drug monitoring in ALL patients, provision of funding, and education activities for professionals in the setting of pediatric‐oncology units are necessary for improving the care of childhood ALL in daily conditions.

## AUTHOR CONTRIBUTIONS

**Alvaro Lassaletta:** Conceptualization (lead); data curation (lead); formal analysis (lead); funding acquisition (lead); investigation (lead); methodology (lead); project administration (lead); validation (lead); visualization (lead); writing – original draft (lead); writing – review and editing (lead). **Fernando Gutiérrez Nicolás:** Conceptualization (lead); data curation (lead); formal analysis (lead); funding acquisition (lead); investigation (lead); methodology (lead); project administration (lead); supervision (lead); validation (lead); writing – original draft (lead); writing – review and editing (lead).

## CONFLICT OF INTEREST

The authors declare that there no conflict of interest with respect to this study.

## ETHICS STATEMENT

Since no patient data were collected, the study did not need to be submitted to a clinical research ethics committee and was exempt from the requirement for informed consent.

## Data Availability

The data used to support the findings of this study are available from the corresponding author upon request.

## APPENDIX A Investigators of the PEGASO study

Hermenegildo González García, Jaime Verdú Amorós, Alejandra Regueiro García, Carmen Rodríguez‐Vigil, Soledad González Muñiz, Monica López Duarte, Encarnación Villar Quesada, María Tasso Cereceda, Miren Oscoz Lizarbe, Antonio Molinés Honrubia, Miriam García Abós, María Tallón García, Susana Riesco Riesco, José Luis Fuster Soler, Marina García Morin, Blanca Herrero Velasco, Inmaculada Marchante Cepillo, Ágeda Molinos Quintana, Carlos Alcaide Álvarez, Victor Quintero Calcaño, Berta González Martínez, Macarena González Cruz, Ana Belén Alas, Antonia Rodríguez Villa, Marta Baragaño González, María Baro Fernández, Ana Fernández‐Teijeiro Álvarez, Ana I. Rodríguez Jiménez, Thais Murciano Carrillo, José María Fernández Navarro, Emilia Urrutia Maldonado, José Manuel Vagace Valero, Francisco Lendínez Molinos, Jimena De Pedro Olabarri, Laia Ferrés Ramis, Montserrat Mesegué Meda, Eva Gálvez De La Villa, Carlos Alcaide Álvarez, María Del Pozo Carlavilla.

## References

[1] SEER Cancer Statistics Review (CSR) 1975‐2018. Released April 15, 2021. Available at: https://seer.cancer.gov/csr/1975_2018/. Accessed November 8, 2021.

[2] American Cancer Society . Key statistics for acute lymphocytic leukemia (ALL). Available at: https://www.cancer.org/cancer/acute-lymphocytic-leukemia/about/key-statistics.html. Accessed November 8, 2021.

[3] Leach L. Relapse in acute lymphoblastic leukemia (ALL). A Guide for Patients Available at: https://media.leukemiacare.org.uk. Accessed November 8, 2021.

[4] Butler M , van der Meer LT , van Leeuwen FN . Amino acid depletion therapies: starving cancer cells to death. Trends Endocrinol Metab. 2021;32(6):367‐381. 33795176 10.1016/j.tem.2021.03.003

[5] Chiu M , Taurino G , Bianchi MG , Kilberg MS , Bussolati O . Asparagine synthetase in cancer: beyond acute lymphoblastic leukemia. Front Oncol. 2020;9:1480. doi:10.3389/fonc.2019.01480 31998641 PMC6962308

[6] Paillassa J , Leguay T , Thomas X , et al. Monitoring of asparagine depletion and anti‐L‐asparaginase antibodies in adult acute lymphoblastic leukemia treated in the pediatric‐inspired GRAALL‐2005 trial. Blood Cancer J. 2018;8(5):45. doi:10.1038/s41408-018-0084-5 29795175 PMC5966449

[7] Jarrar M , Gaynon PS , Periclou AP , et al. Asparagine depletion after pegylated *Escherichia coli* asparaginase treatment and induction outcome in children with acute lymphoblastic leukemia in first bone marrow relapse: a children's oncology group study (CCG‐1941). Pediatr Blood Cancer. 2006;47(2):141‐146. 16425271 10.1002/pbc.20713

[8] Wetzler M , Sanford BL , Kurtzberg J , et al. Effective asparagine depletion with pegylated asparaginase results in improved outcomes in adult acute lymphoblastic leukemia: cancer and leukemia group B study 9511. Blood. 2007;109(10):4164‐4167. 17264295 10.1182/blood-2006-09-045351 PMC1885493

[9] Panosyan EH , Seibel NL , Martin‐Aragon S , et al. Asparaginase antibody and asparaginase activity in children with higher‐risk acute lymphoblastic leukemia: Children's cancer group study CCG‐1961. J Pediatr Hematol Oncol. 2004;26(4):217‐226. 15087948 10.1097/00043426-200404000-00002

[10] Tong WH , Pieters R , Kaspers GJ , et al. A prospective study on drug monitoring of PEGasparaginase and *Erwinia* asparaginase and asparaginase antibodies in pediatric acute lymphoblastic leukemia. Blood. 2014;123(13):2026‐2033. 24449211 10.1182/blood-2013-10-534347 PMC3968389

[11] Würthwein G , Lanvers‐Kaminsky C , Gerss J , et al. Therapeutic drug monitoring of asparaginase: intra‐individual variability and predictivity in children with acute lymphoblastic leukemia treated with PEG‐asparaginase in the AIEOP‐BFM acute lymphoblastic leukemia 2009 study. Ther Drug Monit. 2020;42(3):435‐444. 32022785 10.1097/FTD.0000000000000727

[12] Vieira Pinheiro JP , Müller HJ , Schwabe D , et al. Drug monitoring of low‐dose PEG‐asparaginase (Oncaspar) in children with relapsed acute lymphoblastic leukaemia. Br J Haematol. 2001;113(1):115‐119. 11328290 10.1046/j.1365-2141.2001.02680.x

[13] Wenner KA , Vieira Pinheiro JP , Escherich G , et al. Asparagine concentration in plasma after 2,500 IU/m(2) PEG‐asparaginase i.v. in children with acute lymphoblastic leukemia. Klin Padiatr. 2005;217(6):321‐326. 16307417 10.1055/s-2005-872516

[14] van der Sluis IM , Vrooman LM , Pieters R , et al. Consensus expert recommendations for identification and management of asparaginase hypersensitivity and silent inactivation. Haematologica. 2016;101(3):279‐285. 26928249 10.3324/haematol.2015.137380 PMC4815719

[15] Mondelaers V , Ferster A , Uyttebroeck A , et al. Prospective, real‐time monitoring of pegylated Escherichia coli and *Erwinia* asparaginase therapy in childhood acute lymphoblastic leukaemia and non‐Hodgkin lymphoma in Belgium. Br J Haematol. 2020;190(1):105‐114. 32057100 10.1111/bjh.16495

[16] Hernández‐Marqués C , Andión M , Perez‐Somarriba M , Madero L , Lassaletta A . Can monitoring asparaginase activity help us to manage toxicity in pediatric acute lymphoblastic leukemia? Leuk Lymphoma. 2020;61(4):990‐992. 31749392 10.1080/10428194.2019.1691191

[17] Asselin B , Rizzari C . Asparaginase pharmacokinetics and implications of therapeutic drug monitoring. Leuk Lymphoma. 2015;56(8):2273‐2280. 25586605 10.3109/10428194.2014.1003056 PMC4732456

[18] Baruchel A , Brown P , Rizzari C , et al. Increasing completion of asparaginase treatment in childhood acute lymphoblastic leukemia (ALL): summary of an expert panel discussion. ESMO Open. 2020;5(5):e000977. doi:10.1136/esmoopen-2020-000977 32967920 PMC7513670

[19] Pike M , Kulkarni K , MacDonald T . Asparaginase activity monitoring and management of asparaginase hypersensitivity reactions in Canada. J Oncol Pharm Pract. 2021. doi:10.1177/10781552211055405. 34854779

[20] Cecconello DK , Werlang ICR , Alegretti AP , et al. Monitoring asparaginase activity in middle‐income countries. Lancet Oncol. 2018;19(9):1149‐1150. 30078698 10.1016/S1470-2045(18)30584-9

[21] Pui CH , Cheng C , Leung W , et al. Extended follow‐up of long‐term survivors of childhood acute lymphoblastic leukemia. N Engl J Med. 2003;349(7):640‐649. 12917300 10.1056/NEJMoa035091

[22] Devilli L , Garonzi C , Balter R , et al. Long‐term and quality of survival in patients treated for acute lymphoblastic leukemia during the pediatric age. Hematol Rep. 2021;13(1):8847. doi:10.4081/hr.2021.8847 33747412 PMC7967269

[23] Safary A , Moniri R , Hamzeh‐Mivehroud M , Dastmalchi S . Highly efficient novel recombinant L‐asparaginase with no glutaminase activity from a new halo‐thermotolerant bacillus strain. Bioimpacts. 2019;9(1):15‐23. 30788256 10.15171/bi.2019.03 PMC6378094

[24] Rau RE , Dreyer Z , Choi MR , et al. Outcome of pediatric patients with acute lymphoblastic leukemia/lymphoblastic lymphoma with hypersensitivity to pegaspargase treated with PEGylated *Erwinia* asparaginase, pegcrisantaspase: a report from the Children's oncology group. Pediatr Blood Cancer. 2018;65(3):e26873. doi:10.1002/pbc.26873 PMC5839116 29090524

[25] Sidhu J , Masurekar AN , Gogoi MP , et al. Activity and toxicity of intramuscular 1000 iu/m(2) polyethylene glycol‐E. coli L‐asparaginase in the UKALL 2003 and UKALL 2011 clinical trials. Br J Haematol. 2022;198:142‐150. 35348200 10.1111/bjh.18158 PMC9314843

[26] Medawar CV , Mosegui GBG , Vianna CMM , Costa TMAD . PEG‐asparaginase and native *Escherichia coli* L‐asparaginase in acute lymphoblastic leukemia in children and adolescents: a systematic review. Hematol Transfus Cell Ther. 2020;42(1):54‐61. 31412986 10.1016/j.htct.2019.01.013 PMC7031090

[27] Dinndorf PA , Gootenberg J , Cohen MH , Keegan P , Pazdur R . FDA drug approval summary: pegaspargase (oncaspar) for the first‐line treatment of children with acute lymphoblastic leukemia (ALL). Oncologist. 2007;12(8):991‐998. 17766659 10.1634/theoncologist.12-8-991

[28] Brigitha LJ , Pieters R , van der Sluis IM . How much asparaginase is needed for optimal outcome in childhood acute lymphoblastic leukemia? Syst Rev Eur J Cancer. 2021;157:238‐249. 10.1016/j.ejca.2021.08.025 34536947

[29] Pieters R , Hunger SP , Boos J , et al. L‐asparaginase treatment in acute lymphoblastic leukemia: a focus on *Erwinia* asparaginase. Cancer. 2011;117(2):238‐249. 20824725 10.1002/cncr.25489 PMC3000881

[30] Maese L , Loh ML , Lin T , et al. Initial results from a phase 2/3 study of recombinant *Erwinia* asparaginase (JZP458) in patients with acute lymphoblastic leukemia (ALL)/lymphoblastic lymphoma (LBL) who are allergic/hypersensitive to *E. coli*‐derived asparaginases. Blood. 2021;138(1):2307.34882216

[31] Egler RA , Ahuja SP , Matloub Y . L‐asparaginase in the treatment of patients with acute lymphoblastic leukemia. J Pharmacol Pharmacother. 2016;7(2):62‐71. 27440950 10.4103/0976-500X.184769 PMC4936081

[32] Boos J , Werber G , Ahlke E , et al. Monitoring of asparaginase activity and asparagine levels in children on different asparaginase preparations. Eur J Cancer. 1996;32A(9):1544‐1550. 8911116 10.1016/0959-8049(96)00131-1

[33] Schrey D , Borghorst S , Lanvers‐Kaminsky C , et al. Therapeutic drug monitoring of asparaginase in the ALL‐BFM 2000 protocol between 2000 and 2007. Pediatr Blood Cancer. 2010;54(7):952‐958. 20108339 10.1002/pbc.22417

[34] Marini BL , Brown J , Benitez L , et al. A single‐center multidisciplinary approach to managing the global *Erwinia* asparaginase shortage. Leuk Lymphoma. 2019;60(12):2854‐2868. 31099289 10.1080/10428194.2019.1608530

[35] McCormick M , Lapinski J , Friehling E , Smith K . Premedication prior to PEG‐asparaginase is cost‐effective in pediatric patients with acute lymphoblastic leukemia. Pediatr Blood Cancer. 2021;68(8):e29051. doi:10.1002/pbc.29051 33860989

[36] Cooper SL , Young DJ , Bowen CJ , Arwood NM , Poggi SG , Brown PA . Universal premedication and therapeutic drug monitoring for asparaginase‐based therapy prevents infusion‐associated acute adverse events and drug substitutions. Pediatr Blood Cancer. 2019;66(8):e27797. doi:10.1002/pbc.27797 31099154 PMC8294186

